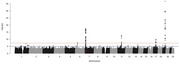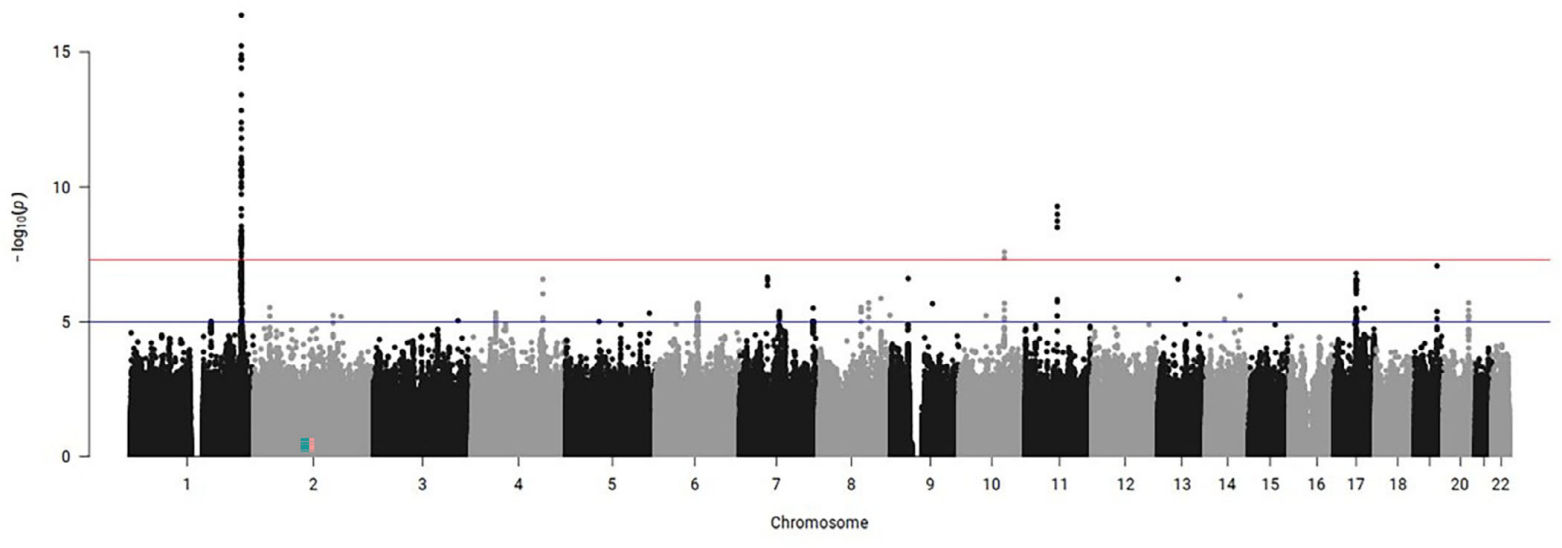# Multi‐ancestry genome‐wide association study of plasma glial fibrillary acidic protein (GFAP)

**DOI:** 10.1002/alz.091361

**Published:** 2025-01-09

**Authors:** Myriam Fornage, Rui Xia, Marcio A Almeida, Joshua C Bis, Biqi Cui, Itziar de Rojas, Charles Decarli, Valborg Gudmundsdottir, Xueqiu Jian, Aniket Mishra, Thomas H. Mosley, Clàudia Olivé, Qiong Yang, Pascual Sanchez‐Juan, John Blangero, Stéphanie Debette, Hector M González, Vilmundur Gudnason, Lenore J. Launer, Bruce M. Psaty, Agustin Ruiz, Claudia L Satizabal, Sudha Seshadri

**Affiliations:** ^1^ University of Texas Health Science Center at Houston, Houston, TX USA; ^2^ University of Texas Rio Grande Valley, Brownsville, TX USA; ^3^ University of Washington, Seattle, WA USA; ^4^ The University of Texas Health Science Center at San Antonio, San Antonio, TX USA; ^5^ Central South University, Changsha, Hunan China; ^6^ Fundació ACE, Institut Català de Neurociències Aplicades, Barcelona Spain; ^7^ University of California, Davis, Sacramento, CA USA; ^8^ University of Iceland, Reykjavik Iceland; ^9^ University of Bordeaux, Bordeaux France; ^10^ University of Mississippi Medical Center, Jackson, MS USA; ^11^ Universitat Oberta de Catalunya, Barcelona Spain; ^12^ Boston University, Boston, MA USA; ^13^ CIEN Foundation/Queen Sofia Foundation Alzheimer Center, Madrid Spain; ^14^ University of Texas Rio Grande Valley School of Medicine, Brownsville, TX USA; ^15^ University of California, San Diego, La Jolla, CA USA; ^16^ Icelandic Heart Association, Kopavogur Iceland; ^17^ National Institute on Aging, Baltimore, MD USA

## Abstract

**Background:**

Glial fibrillary acidic protein (GFAP) is an astrocytic cytoskeletal protein and a promising blood biomarker for Alzheimer's disease (AD) and other neurodegenerative diseases. To date, the genetic architecture of plasma GFAP has not been characterized. We conducted a multi‐ancestry meta‐analyses of genome‐wide association studies (GWAS) in diverse population‐based cohorts to identify genetic variants associated with plasma levels of GFAP and to investigate their implication for neurological diseases.

**Methods:**

Circulating GFAP levels were assayed by ultrasensitive single molecule array (Simoa) immunoassay. We performed ancestry‐specific and ancestry‐combined meta‐analyses of GWAS in 17,028 individuals (mean age: 64.5 years; 60% women) of diverse ancestry (African (AA), N=1163; Hispanic (HA), N=7405; European (EA), N=8460) from 12 community‐based cohorts.

**Results:**

In the combined sample, we identified 7 loci associated with plasma GFAP. These were located in or near genes encoding APOE, CAPN1, CAPN2, GFAP, TMEM106B, PPP1R3C, and LOC105375672. The strongest association was with the APOE4 variant rs429358 (P=2.6x10^‐40^). However, this association was heterogeneous by ancestry, with stronger effects in EA, weaker in HA, and no association in AA. Ancestry‐specific meta‐analyses identified additional loci, including ZFAND3 in EA (Figure 1) and CAPN2 in HA (Figure 2), which were not observed in the other ancestries. No locus reached genome‐wide significance in AA. Using fine‐mapping methods incorporating functional annotation, gene expression, and linkage disequilibrium, we further prioritized potential causal variants and genes at the identified loci and investigated their implication for major neurological diseases, including AD.

**Conclusion:**

Our GWAS of plasma GFAP has identified multiple loci with ancestry‐specific or cross‐ancestry effects. Our multi‐ancestry, integrative approach extends knowledge about the biology of GFAP and its shared genetic architecture with neurological disease.